# Long-term study of the safety and maintenance of efficacy of solriamfetol (JZP-110) in the treatment of excessive sleepiness in participants with narcolepsy or obstructive sleep apnea

**DOI:** 10.1093/sleep/zsz220

**Published:** 2019-11-06

**Authors:** Atul Malhotra, Colin Shapiro, Jean-Louis Pepin, Jaz Hedner, Mansoor Ahmed, Nancy Foldvary-Schaefer, Patrick J Strollo, Geert Mayer, Kathleen Sarmiento, Michelle Baladi, Patricia Chandler, Lawrence Lee, Richard Schwab

**Affiliations:** 1 Division of Pulmonary, Critical Care and Sleep Medicine, University of California San Diego, La Jolla; 2 University of Toronto, ON, Canada; 3 HP2 Laboratory, INSERM U1042, University Grenoble Alpes, France; 4 EFCR Laboratory, Pole Thorax et Vaisseaux, Grenoble Alpes University Hospital, France; 5 Sahlgrenska University Hospital, Gothenburg University, Sweden; 6 Cleveland Sleep Research Center, OH; 7 Cleveland Clinic Lerner College of Medicine, OH; 8 University of Pittsburgh/Veterans Administration Pittsburgh Health System, PA; 9 Hephata Klinik, Schwalmstadt, Germany; 10 Philipps University, Marburg, Germany; 11 San Francisco Veterans Administration Healthcare System, CA; 12 Jazz Pharmaceuticals, Palo Alto, CA; 13 University of Pennsylvania, Philadelphia

**Keywords:** solriamfetol, JZP-110, excessive sleepiness, narcolepsy, obstructive sleep apnea

## Abstract

**Study Objectives:**

To evaluate long-term safety and maintenance of efficacy of solriamfetol treatment for excessive daytime sleepiness in narcolepsy and obstructive sleep apnea (OSA).

**Methods:**

Participants with narcolepsy or OSA who completed a prior solriamfetol study were eligible. A 2-week titration period was followed by a maintenance phase (up to 50 weeks). Efficacy was assessed by Epworth Sleepiness Scale (ESS) and Patient and Clinical Global Impression of Change (PGI-C and CGI-C, respectively). After approximately 6 months of treatment, a subgroup entered a 2-week placebo-controlled randomized withdrawal (RW) phase. Change in ESS from beginning to end of the RW phase was the primary endpoint; PGI-C and CGI-C were secondary endpoints. Safety was assessed throughout the study.

**Results:**

In the maintenance phase, solriamfetol-treated participants demonstrated clinically meaningful improvements on ESS, PGI-C, and CGI-C. In the RW phase, least squares mean change on ESS was 1.6 in participants continuing solriamfetol versus 5.3 in participants switched to placebo (*p* < .0001). For both secondary endpoints, higher percentages of participants receiving placebo were reported as worse at the end of the RW phase versus solriamfetol (*p* < .0001). Common treatment-emergent adverse events (TEAEs) with solriamfetol were headache, nausea, nasopharyngitis, insomnia, dry mouth, anxiety, decreased appetite, and upper respiratory tract infection; 27 (4.2%) participants experienced at least one serious TEAE, and 61 (9.5%) withdrew because of TEAEs.

**Conclusions:**

This study demonstrated long-term maintenance of efficacy of solriamfetol under open-label and double-blind, placebo-controlled conditions. Safety profile of solriamfetol was consistent with previous 12-week studies; no new safety concerns were identified.

**Trial Registration:**

NCT02348632

**Statement of Significance**

This long-term study of solriamfetol of up to 1 year, which included a placebo-controlled, double-blind, randomized withdrawal phase after approximately 6 months of open-label treatment, was a follow-up to shorter-term randomized clinical trials of solriamfetol for the treatment of excessive daytime sleepiness (EDS) in adults with narcolepsy or obstructive sleep apnea (OSA). Results from this study provide well-controlled data that demonstrated long-term maintenance of efficacy, with improvements in EDS that were clinically relevant, and a safety profile consistent with shorter-term clinical studies. These results suggest that solriamfetol represents an effective treatment for EDS in adults with narcolepsy and OSA.

## Introduction

In narcolepsy and obstructive sleep apnea (OSA), excessive daytime sleepiness (EDS) is a prominent symptom that is associated with adverse consequences including reductions in function and daily activities, poor health-related quality of life, reduction in work productivity, and increased risk of workplace and driving accidents [1–4]. OSA, which may be present in up to 38% of the general population [5], is characterized by repetitive collapse of the pharyngeal airway leading to intermittent hypoxemia and sleep fragmentation with cardiovascular, metabolic, and cognitive sequelae that result in increased morbidity and mortality [6]. Nasal continuous positive airway pressure (CPAP) is the treatment of choice for OSA, but is associated with nonadherence in more than one-third of patients who initiate such therapy [7]. In addition, despite adequate treatment of the underlying airway obstruction by CPAP and other primary therapies for OSA, EDS is estimated to persist in 12%–65% of patients, even with adherence to primary therapy [8–12]. Narcolepsy is a less common disorder than OSA that results from abnormal function of the sleep–wake neuronal pathways, and is characterized in patients with type 1 narcolepsy by orexin deficiency [13]. EDS is a defining characteristic of narcolepsy, and the degree of this sleepiness is severe in most patients [14].

Although EDS is not often diagnosed or treated as a condition in and of itself, several pharmacologic therapies including stimulants and wake-promoting agents are available for the treatment of EDS [15–17]. Amphetamines are considered an effective approach to treatment, although there are no well-controlled studies demonstrating their efficacy and safety profile in narcolepsy [18]. In the United States, modafinil and armodafinil are approved to improve wakefulness in patients with narcolepsy, OSA, and shift work disorder [19, 20], with data from short-term randomized controlled trials and long-term, open-label studies showing efficacy for the treatment of EDS in those conditions [21–29]. Although modafinil and armodafinil clearly have efficacy, there are some patients in whom there may be a lack of efficacy throughout the day [18, 25, 30]. In addition, modafinil is not available in many countries, including limited use in the European community, where it is no longer available for the treatment of OSA due to concerns about its unfavorable benefit/risk profile [31]. Moreover, modafinil and armodafinil are primarily eliminated via metabolism, mainly in the liver, and both have drug interactions that include reduced efficacy of oral contraceptives [19, 20].

Solriamfetol (formerly JZP-110), a dopamine and norepinephrine reuptake inhibitor with no significant monoamine-releasing effects [32], has been approved in the United States to improve wakefulness in adult patients with EDS associated with narcolepsy or OSA [33]. The approved dose range of solriamfetol in the United States is 75–150 mg once daily for patients with narcolepsy and 37.5–150 mg once daily for patients with OSA [33]. In randomized clinical trials, solriamfetol demonstrated efficacy for the treatment of EDS in patients with narcolepsy and OSA [34–38]. However, these studies were for a maximum of 12 weeks, and did not evaluate the long-term efficacy and safety of this agent.

Given that narcolepsy and OSA are chronic health conditions, it is important to evaluate whether well-controlled data support the long-term use of solriamfetol in these diseases. Thus, the aim of this study was to evaluate the long-term safety and maintenance of efficacy, including the inclusion of a double-blind, placebo-controlled randomized withdrawal (RW) period, of solriamfetol in the treatment of EDS in adults with narcolepsy or OSA.

## Methods

### Study design

**Figure 1.**
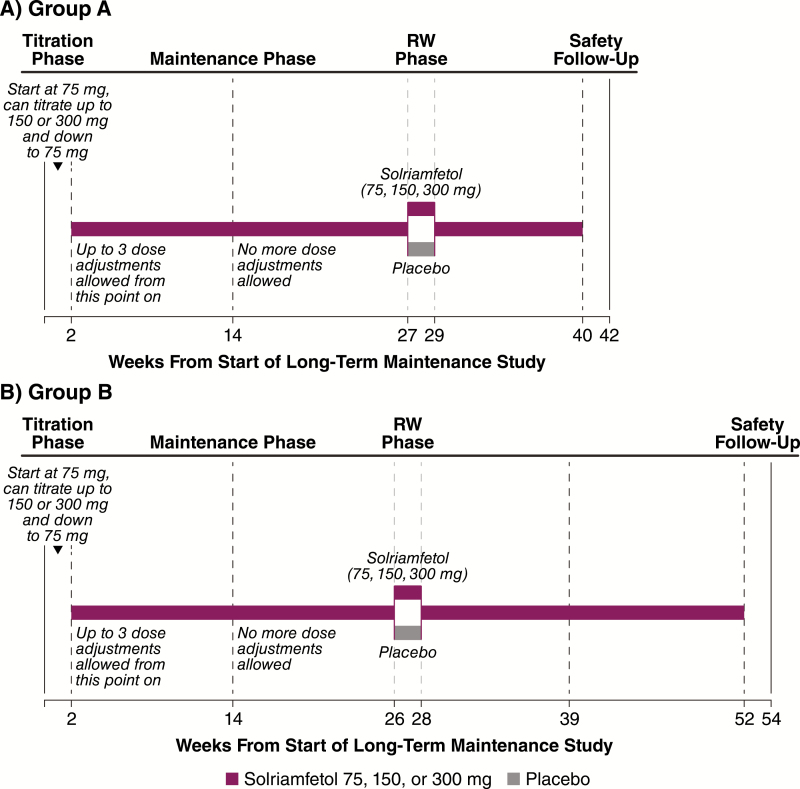
Study design. RW, randomized withdrawal.

The long-term safety and maintenance of efficacy of solriamfetol were evaluated in participants with narcolepsy or OSA who had previously completed a clinical trial of solriamfetol [34–38]. A 2-week titration phase was followed by a maintenance phase of up to 50 weeks (Figure 1). After approximately 6 months of open-label treatment with solriamfetol, a subgroup of patients entered a 2-week placebo-controlled RW phase (Figure 1), and the maintenance phase was resumed after RW phase completion.

The study was approved by institutional review boards or ethics committees at each site and was performed in accordance with the Declaration of Helsinki; all participants provided written informed consent (www.clinicaltrials.gov identifier NCT02348632, and https://eudract.ema.europa.eu/ #2014-005489-31).

### Participants

Eligible participants were individuals with narcolepsy or OSA who had previously completed a phase 2 or 3 clinical trial of solriamfetol [34–38]. Owing to differences in study design as well as variable duration between prior study completion and enrollment in the long-term study, participants were enrolled into one of two groups. Group A included participants who completed a phase 3, 12-week narcolepsy (NCT02348593) [36] or OSA (NCT02348606) [37] study, and who immediately enrolled into this long-term study; the study duration in this group was for 40 weeks. Group B included participants with narcolepsy or OSA who completed one of the phase 2 studies (NCT01485770, NCT01681121, NCT02806908, and NCT02806895) [34, 35] or the 6-week, phase 3 study (NCT02348619) [38] and were subsequently enrolled into this long-term study. These participants had a study duration for 52 weeks.

In addition to having completed a prior study with solriamfetol, the other inclusion criteria were similar to those previously reported for the parent studies, and included that participants were required to have usual nightly sleep of at least 6 hours, and a body mass index of 18 to less than 45 kg/m^2^. Participants in the OSA studies were those with current or prior use of a primary OSA therapy; participants without current primary OSA therapy use or a history of a surgical intervention for OSA were required to have tried to use a primary OSA therapy for at least 1 month with at least one documented adjustment to the therapy. Exclusion criteria were similar to those previously reported for the parent studies, and included characteristics either associated with EDS or that could affect evaluation of EDS [34–38]. In addition, patients in previous studies who experienced any serious adverse event (AE) that was deemed related to solriamfetol or a treatment-emergent adverse event (TEAE) that might prevent safe participation in this study were excluded. Use of any over-the-counter or prescription medication (e.g. stimulants, modafinil, sodium oxybate) that could affect the evaluation of EDS were prohibited. However, in participants with narcolepsy, use of anticataplectic medications was allowed, including selective serotonin reuptake inhibitors, serotonin–norepinephrine reuptake inhibitors, and tricyclic antidepressants.

### Treatment

During the 2-week titration phase, participants began with a once-daily dose of 75 mg and were able to titrate up one dose level once every 3 days (to 150 mg/d and then a maximum dose of 300 mg/d) (Figure 1). Participants were also able to titrate down to 75 or 150 mg at any time. Investigators were instructed to titrate to a maximal tolerated dose. The titration phase was followed by a maintenance phase for a total duration of 40 weeks in group A and 52 weeks in group B. After the titration phase, only three additional dose adjustments were allowed during the first 12 weeks of the maintenance phase.

After approximately 6 months of open-label treatment, 282 participants (203 with OSA and 79 with narcolepsy), representing a subgroup comprising patients from group A and group B, were randomized into the 2-week, double-blind, placebo-controlled RW phase; randomization was initiated and continued until the number of participants was met based on the power calculation described below in the Statistical Analysis section. At the beginning of this phase, participants were randomly assigned in a 1:1 ratio, stratified by condition, to continue to receive solriamfetol at the dose that they were currently receiving or to receive placebo for 2 weeks. All study personnel and participants were blinded to the study treatments during the RW phase, with study drugs prepared in identical opaque gelatin capsules to ensure adequate blinding; the same type of gelatin capsules were used in the maintenance phase. At the end of the RW phase, participants received the same dose of solriamfetol they had been receiving at the beginning of the RW phase for the remainder of the study, with a fixed titration of 3 days for participants on the 150- and 300-mg doses (i.e. participants who had been receiving 150 mg/d received 75 mg/d for the first 3 days, followed by 150 mg/d thereafter, and participants who had been receiving 300 mg/d received 150 mg/d for the first 3 days, followed by 300 mg/d thereafter).

### Efficacy measures

During the maintenance phase with open-label treatment of solriamfetol, efficacy was assessed at the end of the 2-week titration phase and at approximately 14, 27, 29, and 40 weeks after the start of treatment in group A, and at approximately 14, 26, 28, 39, and 52 weeks after the start of treatment in group B. Efficacy assessments included Epworth Sleepiness Scale (ESS) [39], and percentage of participants with any improvement (minimally, much, or very much) as reported by the study participants on the Patient Global Impression of Change (PGI-C) and by the clinician on the Clinical Global Impression of Change (CGI-C) [40].

The ESS is a validated patient-reported outcome that assesses the propensity to fall asleep in different situations; the score range is 0–24, with higher scores indicating greater sleepiness and scores less than or equal to 10 considered to be within the normal range [39, 41]. The PGI-C and the CGI-C are both assessed on a 7-point scale (1 = very much improved to 7 = very much worse) [40].

In the RW phase, the primary efficacy endpoint was change in ESS from the beginning to the end of the 2-week RW phase. Secondary endpoints were the percentages of participants with any worsening (minimally, much, or very much) on the PGI-C and CGI-C at the end of the RW phase.

### Safety

The safety and tolerability of solriamfetol were evaluated across the entire study, and consisted of TEAEs and assessment of clinical laboratory tests, electrocardiograms, vital signs, and the Columbia-Suicide Severity Rating Scale (C-SSRS) [42]. The potential for withdrawal effects after solriamfetol was explored after abrupt discontinuation that occurred when participants received placebo during the double-blind, RW period. Withdrawal-related AEs were preselected based on validated instruments, such as the Amphetamine Cessation Symptom Assessment scale [43], Amphetamine Withdrawal Questionnaire [44], Cocaine Selective Severity Assessment [45], and symptoms of stimulant withdrawal from the *Diagnostic and Statistical Manual of Mental Disorders, Fifth Edition* (*DSM-5*) [46].

### Statistical analysis

Descriptive statistics were used for analysis of open-label efficacy, which was conducted on the safety population, defined as all participants who received at least one dose of study medication in this study; no formal statistical testing was performed, and missing data were imputed using a last-observation-carried-forward approach. In addition, a post hoc analysis was conducted, also using a last-observation-carried-forward approach, to determine the percentage of participants in the combined solriamfetol group who had an ESS score in the normal range (less than or equal to 10) at the end of the study. As participants were not randomly assigned to doses but were titrated to their maximal tolerated dose, results are not presented by dose.

As participants in group A were enrolled immediately after completion of the previous randomized controlled trial, data were available for both baseline of the parent study and last assessment of the parent study; the change from baseline of the parent study was the focus of data presentation in the current analysis. For participants in group B, there was a time gap for most participants between completion of the previous randomized controlled trial and enrollment in this study, thus, baseline reflects the first visit in this study.

It was estimated that a sample size of 150 participants per treatment group (placebo, solriamfetol) in the RW phase would provide at least 95% power to detect a difference of 3 points in the ESS score from the beginning to the end of the 2-week RW phase. This calculation assumed a common *SD* of 7 points for the ESS change during the RW phase and a two-sided significance level of 0.05 using a *t*-test. Analyses of the RW phase were conducted on the modified intention-to-treat (mITT) population, defined as participants who were randomized, received at least one dose of study medication, and had evaluable efficacy data at the end of the RW phase. If a participant in the mITT population did not have an assessment for a particular efficacy endpoint, that participant was excluded in the analysis of that endpoint. An analysis of covariance model was used for ESS, with results reported as least squares (LS) means. The PGI-C and CGI-C were analyzed using a chi-square test. Results were analyzed and are presented for the overall population and by indication (narcolepsy and OSA). A fixed hierarchical testing procedure was used to correct for multiplicity in the overall population, starting with ESS and proceeding to PGI-C, and then CGI-C if statistical significance was met (*p* < .05). Because comparisons were for combined solriamfetol doses versus placebo, there were no multiplicity issues with respect to doses.

All analyses were conducted using SAS version 9.3 or higher (SAS Institute, Cary, NC).

## Results

### Population

Participants comprising the overall safety population (*N* = 643) were mostly white (78.7%) with a mean age of 49.3 years; 52.4% were male (Table 1). Comorbid conditions included hypertension (37.6%), hyperlipidemia (15.2%), and type 2 diabetes (14.0%). The percentages of participants who were titrated to 75, 150, and 300 mg were 10.0%, 32.2%, and 57.9%, respectively.

**Table 1.** Demographic and clinical characteristics (safety population)

		Randomized withdrawal phase	
Variable	Maintenance phase (*N* = 643)	Placebo (*n* = 142)	Solriamfetol (*n* = 140)
Age, years, mean (*SD*)	49.3 (14.2)	50.7 (12.1)	50.2 (13.2)
Male, *n* (%)	337 (52.4)	85 (59.9)	76 (54.3)
Race, *n* (%)			
White	506 (78.7)	110 (77.5)	112 (80.0)
Black/African American	109 (17.0)	25 (17.6)	25 (17.9)
Asian	15 (2.3)	5 (3.5)	3 (2.1)
Other	13 (2.0)	2 (1.4)	0
BMI, kg/m^2^, mean (*SD*)	31.7 (5.9)	31.8 (6.0)	31.9 (5.7)
Disease, *n* (%)			
Narcolepsy	226 (35.1)	40 (28.2)	39 (27.9)
OSA	417 (64.9)	102 (71.8)	101 (72.1)

BMI, body mass index; OSA, obstructive sleep apnea.

The safety population included 226 (35.1%) participants with narcolepsy and 417 (64%) with OSA. Of the narcolepsy subpopulation, 114 (50.4%) participants reported having cataplexy at baseline. In the OSA subpopulation, 77.7% of participants were adherent with primary OSA therapy. Among participants with OSA, 13.7% had a medical history of a surgical intervention for OSA. At study baseline, primary OSA therapy was used by 71.5% of OSA participants; of these participants, 93.2% were using PAP at entry into this study, 2.3% were using another type of device as a primary OSA therapy (e.g. neurostimulator or mandibular advancement device), and 5.4% did not specify the type of primary OSA therapy.

Groups A and B consisted of 519 (80.7%) and 124 (19.3%) participants, respectively. Overall, most participants at baseline (in the parent study for group A or at baseline in this study for group B) were either “moderately” (34.2%), “markedly” (35.9%), or “severely” (17.4%) ill on the Clinical Global Impression of Severity scale. Higher percentages of participants with narcolepsy relative to those with OSA were “markedly” (41.2% vs 33.1%) or “severely” ill (23.9% vs 13.9%).

**Figure 2.**
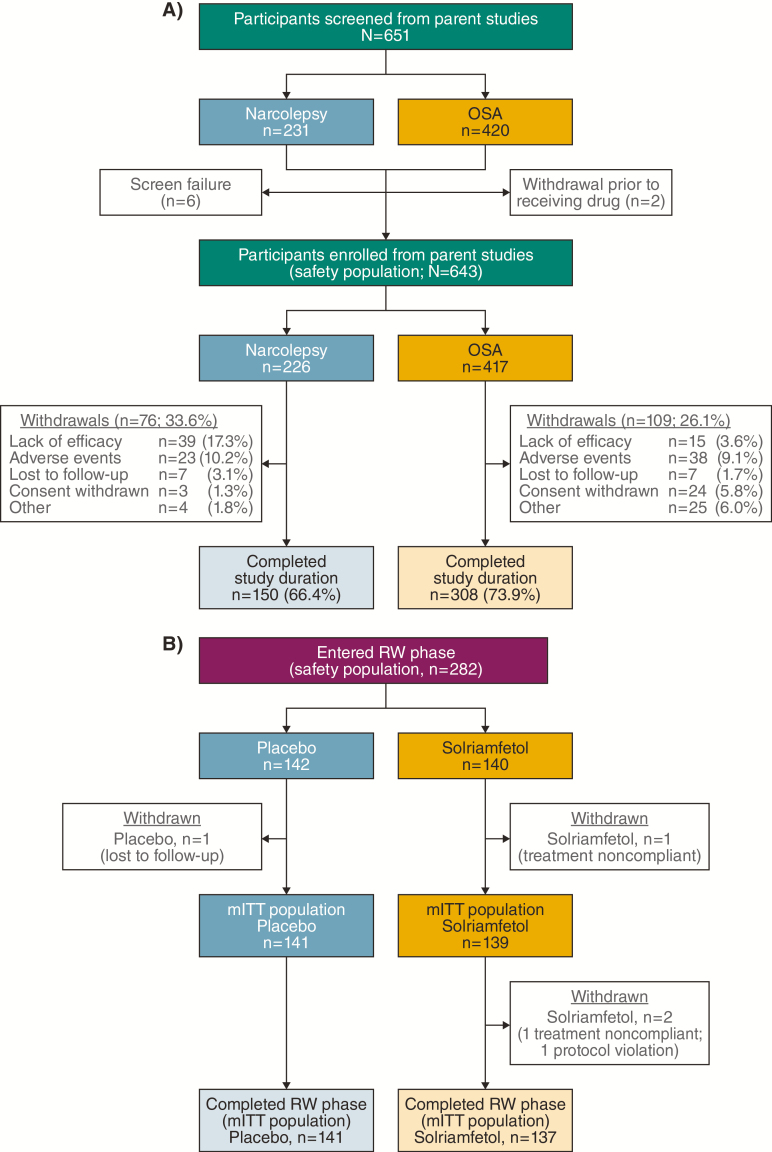
Participant disposition. (A) Maintenance phase. (B) RW phase. mITT, modified intention-to-treat; RW, randomized withdrawal.

A total of 458 participants (71.2%) completed the full study, including 66.4% of narcolepsy participants and 73.9% of OSA participants (Figure 2A). Reasons for withdrawal were comparable between the narcolepsy and OSA subpopulations, with the exception of lack of efficacy, which occurred in 39 of 226 (17.3%) participants with narcolepsy and 15 of 417 (3.6%) participants with OSA. Withdrawal due to TEAEs was observed in 23 of 226 (10.2%) participants with narcolepsy and 38 of 417 (9.1%) participants with OSA.

Baseline demographics and disease characteristics of the 282 participants randomized into the RW period of the study were similar between the two treatment groups, and similar to the overall study population (Table 1). The mITT population of the RW phase consisted of 280 participants who completed this phase; two participants withdrew, both from the solriamfetol group (Figure 2B).

### Efficacy during the maintenance phase

**Figure 3.**
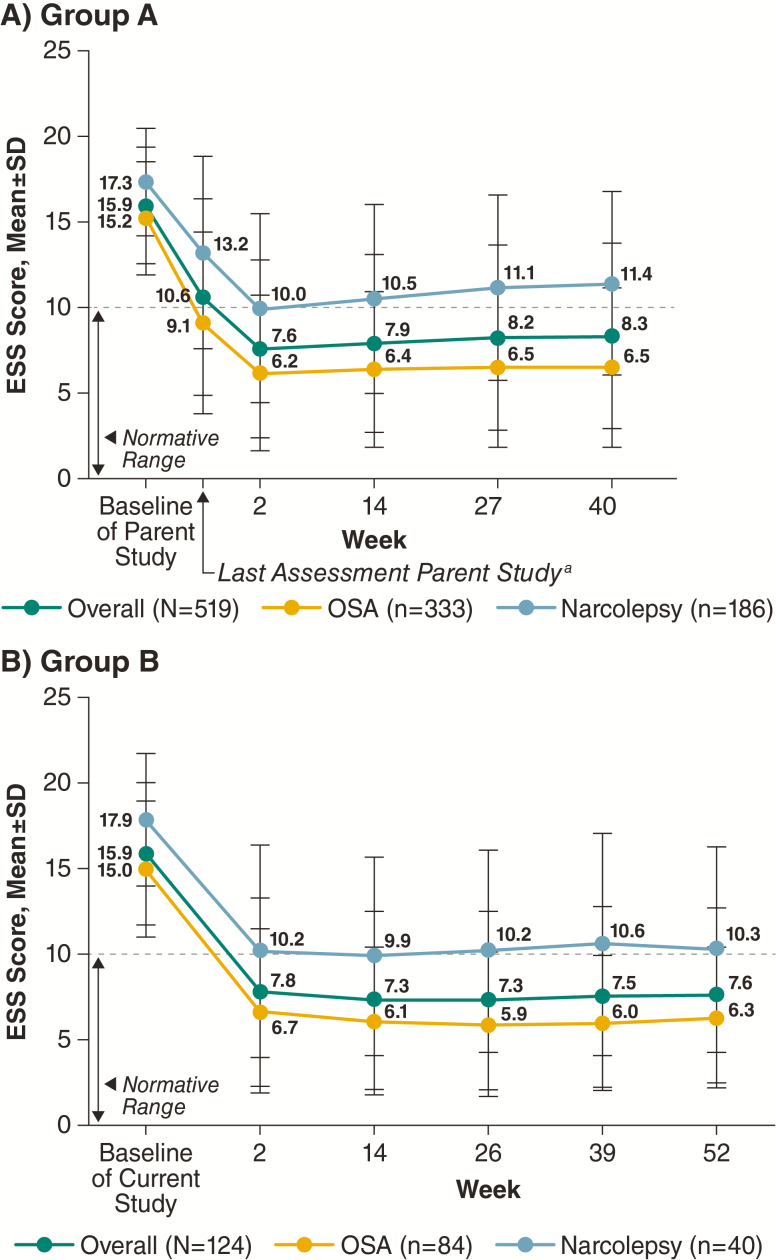
ESS scores during the maintenance phase (safety population). ^a^Not all participants at the last assessment in the parent study were on study drug. ESS, Epworth sleepiness scale; OSA, obstructive sleep apnea.

In the overall population, mean ESS scores were 15.9 for group A and 16.2 for group B at baseline of the parent and current study, respectively. At week 2, mean ESS scores decreased to 7.6 for group A and to 7.8 for group B, and these improvements (i.e. decrease in mean ESS scores) were maintained throughout the study duration (Figure 3). Similar patterns were observed in the individual narcolepsy and OSA populations (Figure 3).

A post hoc analysis showed high percentages of participants had ESS scores less than or equal to 10 at the end of the maintenance phase in both the narcolepsy and OSA populations. In group A, 43.0% of participants with narcolepsy and 81.7% of those with OSA reported ESS scores less than or equal to 10 at week 40, compared with 0.5% and 6.0% of participants with narcolepsy or OSA, respectively, who reported ESS scores less than or equal to 10 at parent study baseline. Similarly, in group B, the percentages were 52.5% for narcolepsy and 84.5% for OSA at week 52 relative to 5.0% and 11.9%, respectively, at current study baseline.

**Figure 4.**
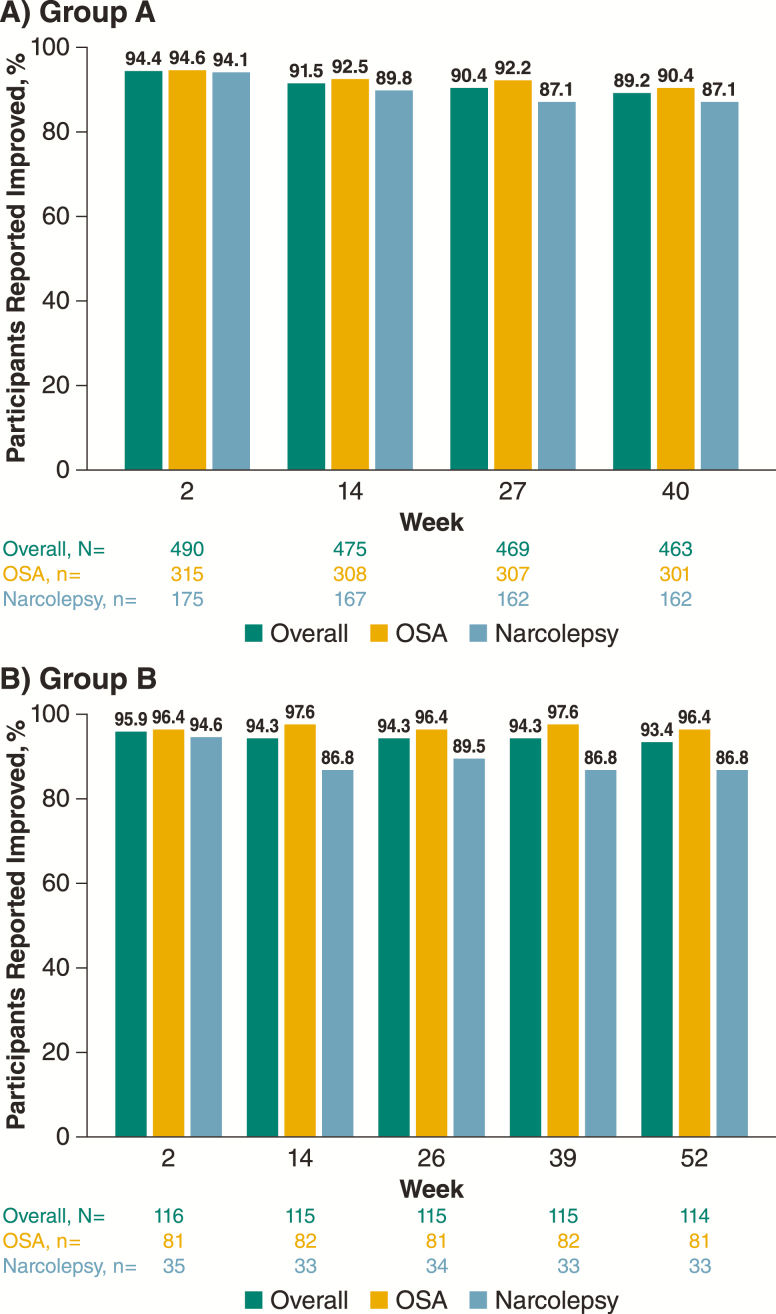
Patient-reported global improvements on PGI-C over time during the maintenance phase (safety population). PGI-C, patient global impression of change; OSA, obstructive sleep apnea.

The majority of participants (>94%) reported improvements on the PGI-C at week 2, and these improvements were maintained at generally similar percentages at each assessment; 87.1%–90.4% of participants in group A and 86.8%–96.4% of participants in group B reported improvement on the PGI-C at the final assessment (Figure 4). Sustained improvements from the first assessment at week 2 over the study duration were also reported from the clinician perspective on the CGI-C, with good concordance with the PGI-C for the percentage of participants who improved (Supplemental Figure S1). Similar patterns were observed in the individual narcolepsy and OSA populations (Figure 4).

### Efficacy during the RW phase

**Figure 5.**
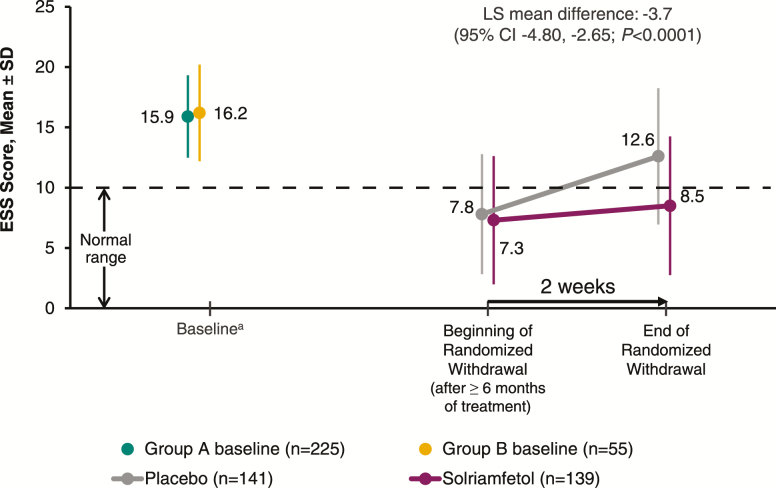
ESS scores among participants who entered the RW phase (mITT population). ^a^Values are for the mITT population at baseline of parent study in participants from group A (*n* = 225) and at baseline of current study in those from group B (*n* = 55); the RW phase included participants from both groups. ESS, Epworth Sleepiness Scale; mITT, modified intention-to-treat; RW, randomized withdrawal.

All primary and secondary endpoints were met for the RW phase (*p* < .0001). Participants who received solriamfetol during the RW phase maintained their improvement from the beginning of the RW phase, whereas those who were randomized to receive placebo worsened (Figure 5). The LS mean change in ESS score was 1.6 with solriamfetol compared with 5.3 with placebo, resulting in a LS mean difference of −3.7 (95% confidence interval −4.80 to −2.65; *p* < .0001).

**Figure 6.**
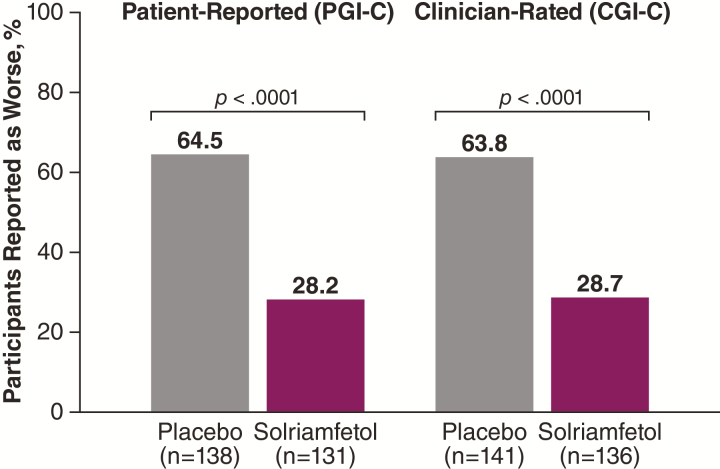
Percentage of participants reported as worse at end of the RW phase (mITT population). CGI-C, clinical global impression of change; mITT, modified intention-to-treat; PGI-C, patient global impression of change; RW, randomized withdrawal.

In the overall population, significantly greater percentages of participants in the placebo group worsened during the RW phase compared with the solriamfetol group on both the PGI-C (64.5% vs 28.2%; *p* < .0001) and CGI-C (63.8% vs 28.7%; *p* < .0001) (Figure 6). Similar results were observed by indication across endpoints (*p* < .05; data not shown).

### Safety

Over the study duration, 482 participants (75%) had at least one TEAE, with similar percentages among those with narcolepsy (74.8%) and OSA (75.1%) (Table 2); 44% of participants (283/643) had a TEAE within the first 2 weeks whereas 12.8% had a TEAE during the second 2 weeks of treatment. Most TEAEs were mild or moderate in severity (Table 2). The most common TEAEs, with a frequency of at least 5% of participants in the combined solriamfetol groups, were headache (11.0%), nausea (8.9%), nasopharyngitis (8.4%), insomnia (7.9%), dry mouth (7.3%), anxiety (7.2%), decreased appetite (5.0%), and upper respiratory tract infection (5.0%) (Table 2). With the exception of sinusitis, nasopharyngitis, and upper respiratory tract infection, the most common TEAEs occurred most often during the first 2 weeks of the study. TEAE profiles were similar in participants with OSA and narcolepsy. During the open-label period, 59 (9.2%) participants had TEAEs that led to withdrawal from the study (Table 2). TEAEs leading to withdrawal most frequently occurred in the system organ classes of psychiatric disorders (*n* = 20; 3.1%), nervous system disorders (*n* = 13; 2.0%), and gastrointestinal disorders (*n* = 8; 1.2%). TEAEs that most frequently led to withdrawal were anxiety (*n* = 7; 1.1%), headache (*n* = 4; 0.6%), insomnia (*n* = 4; 0.6%), irritability (*n* = 4; 0.6%), nausea (*n* = 4; 0.6%), depression (*n* = 3; 0.3%), and dry mouth (*n* = 3; 0.3%).

**Table 2.** TEAEs across the study (safety population, groups A and B combined)

	Number (%) of participants in combined solriamfetol groups		
TEAE	Overall (*N* = 643)	OSA (*n* = 417)	Narcolepsy (*n* = 226)
At least 1 TEAE	482 (75.0)	313 (75.1)	169 (74.8)
Severity of TEAEs			
Mild	188 (29.2)	128 (30.7)	60 (26.5)
Moderate	246 (38.3)	157 (37.6)	89 (39.4)
Severe	48 (7.5)	28 (6.7)	20 (8.8)
Serious TEAEs	27 (4.2)	21 (5.0)	6 (2.7)
TEAEs leading to discontinuation	59 (9.2)	36 (8.6)	23 (10.2)
Death	1 (0.2)*	1 (0.2)	0
Most common TEAEs^†^			
Headache	71 (11.0)	40 (9.6)	31 (13.7)
Nausea	57 (8.9)	31 (7.4)	26 (11.5)
Nasopharyngitis	54 (8.4)	35 (8.4)	19 (8.4)
Insomnia	51 (7.9)	35 (8.4)	16 (7.1)
Dry mouth	47 (7.3)	33 (7.9)	14 (6.2)
Anxiety	46 (7.2)	25 (6.0)	21 (9.3)
Decreased appetite	32 (5.0)	14 (3.4)	18 (8.0)
Upper respiratory tract infection	32 (5.0)	22 (5.3)	10 (4.4)

*Due to sepsis.

^†^≥5% in combined solriamfetol groups for any indication.

OSA, obstructive sleep apnea; TEAE, treatment-emergent adverse event.

Serious TEAEs were reported in 27 participants (4.2%) across all phases, including 21 participants (5.0%) with OSA and 6 participants (2.7%) with narcolepsy (Supplemental Table S1). There was one death due to complications of sepsis in a 70-year-old male with OSA who had a complex medical history (including diabetes mellitus, rheumatoid arthritis, pulmonary fibrosis, coronary artery disease) and whose concomitant medications included two immunosuppressive agents. During his hospitalization for sepsis, he was also diagnosed with a non-ST elevation myocardial infarction thought likely due to demand ischemia associated with sepsis. The death was considered by the investigator to be unrelated to the study drug administration. A total of nine participants, all with OSA, had cardiovascular or potential cardiovascular serious TEAEs: two participants with atrial fibrillation; one each with angina pectoris, chest discomfort, chest pain, noncardiac chest pain, cerebrovascular accident, pulmonary embolism; and one participant with acute myocardial infarction discussed previously. Of these serious TEAEs, two were deemed by the investigator to be related to study drug administration: atrial fibrillation in a participant whose concomitant medications included two types of thyroid medication, and cerebrovascular accident in a participant with a history of hypertension.

Rebound hypersomnia, as assessed by changes on the ESS, was not observed after abrupt discontinuation of solriamfetol in the RW phase. Participants who received placebo for 2 weeks in the RW phase had increased sleepiness that did not exceed baseline levels as measured by the ESS (Figure 5), suggesting a return toward baseline and no rebound hypersomnia.

There was no pattern of withdrawal signs or symptoms based on analysis of AEs that occurred after abrupt discontinuation of long-term exposure to solriamfetol (i.e. the placebo group in the RW phase). During the RW phase, 16 participants (11.3%) in the placebo group had 22 TEAEs and 22 participants (15.7%) in the solriamfetol group had 27 TEAEs. Of these, TEAEs that occurred in the placebo group that may be indicative of withdrawal effects were insomnia, fatigue, somnolence, and asthenia; however, these events either occurred with approximately equal frequency in the placebo and solriamfetol group, or also occurred during the open-label period of the study while participants were on solriamfetol (data not shown).

Vital signs were summarized separately for group A and group B for the maintenance phase (no placebo control). No clinically relevant changes in heart rate (<1 beat per minute [bpm]) or blood pressure (<1 mm Hg) were observed at assessed time points in group A (*n* = 519). However, for group B (*n* = 124), mean increases from baseline ranged from 1.0 to 4.3 mm Hg for systolic blood pressure, 0.8 to 2.4 mm Hg for diastolic blood pressure, and 0.6 to 4.2 bpm for heart rate across the open-label extension (OLE) (up to 52 weeks); these increases were generally greater for participants with narcolepsy relative to OSA (data not shown). No apparent trends were observed to suggest that there were long-term increases (i.e. worsening) in heart rate or blood pressure over time for participants with narcolepsy or OSA (in both group A and group B). During the RW phase, mean decreases in vital signs were generally observed for both the placebo and combined solriamfetol group, and changes were similar between the groups (data not shown). Among the OSA population, no changes in primary OSA therapy device use were observed during the study duration (data not shown).

Six participants had TEAEs potentially associated with motor vehicle accidents or accidents at work during the study duration; four of these six participants were operating a motor vehicle when the accident occurred. Of the four who were driving a vehicle, two were at fault and two were in vehicles that were rear-ended. All six TEAEs were nonserious and all were assessed as not related to study drug with no reports of sleepiness at the time of the accident.

The C-SSRS did not reveal a pattern of suicidal ideation or suicidal behavior related to solriamfetol treatment; there were two positive postbaseline responses on the C-SSRS observed in two participants with histories of depression and associated TEAEs of depression or depressive symptoms. The positive responses were transient in nature and were not reported as TEAEs of suicidal ideation. Although not captured in the C-SSRS, one participant had a TEAE of suicide attempt (i.e. an intentional medication overdose in conjunction with alcohol consumption); this attempt was reported to be in reaction to a negative change in family circumstances.

## Conclusion/Discussion

This study expands on previous 6- and 12-week studies that have demonstrated robust effects of solriamfetol for improvement of EDS associated with narcolepsy and OSA by showing that these effects are maintained for up to 52 weeks of treatment [34–38]. In addition, the double-blind, placebo-controlled maintenance of efficacy data from the RW period provides well-controlled evidence of the long-term efficacy of solriamfetol to treat EDS associated with narcolepsy or OSA. Further, this study provides the first long-term efficacy data on solriamfetol and supports the long-term safety and tolerability profile of this agent. Although both narcolepsy and OSA participants from the prior studies were enrolled, the findings are consistent despite this heterogeneity.

Treatment with solriamfetol resulted in reductions from baseline in participant-reported EDS, as manifested by lower scores on the ESS at the earliest evaluated time point (2 weeks), and these scores were maintained over the study duration. This maintenance of efficacy over time was consistent in the narcolepsy and OSA subpopulations, with no evidence of tolerance. Moreover, the mean ESS scores reported by the participants with OSA were within the normal range (less than or equal to 10) over the entire study duration, and approached the normal range among participants with narcolepsy. In this regard, it should be noted that participants with narcolepsy had higher baseline ESS scores than OSA participants and that the magnitude of reduction in ESS scores at all assessments was similar in the two subpopulations. However, the difference in baseline ESS scores between the two indications, indicative of more severe EDS among those with narcolepsy, may also account, at least in part, for the higher percentage of participants with narcolepsy who withdrew due to lack of efficacy (17.3%) relative to OSA (3.6%). In addition, at end of study, 43%–85% of participants reported ESS scores within the normal range (less than or equal to 10) in the narcolepsy and OSA populations, respectively.

Long-term maintenance of efficacy and the clinical meaningfulness of the changes on the ESS were demonstrated by the consistently high percentage of participants and clinicians who reported improvement on the PGI-C or CGI-C, respectively. There was good concordance between these two scales at all evaluated time points, suggesting that participants and clinicians had similar perceptions of improvement, which were clinically meaningful.

The inclusion of the double-blind, placebo-controlled RW phase provided further support for the maintenance of efficacy of solriamfetol by demonstrating that discontinuation of treatment resulted in worsening on the ESS and the global improvement scales overall and in each indication separately. The RW phase also provides evidence that improvements observed in this study are not simply related to changes that might have occurred over time (e.g. a resolution of symptoms) and that the beneficial effects observed with solriamfetol are not likely to be related to placebo or Hawthorne effects from being in the trial. In addition, there were no signs of rebound effects or withdrawal after long-term use of solriamfetol.

With long-term treatment, the safety and tolerability profiles were consistent with what has previously been reported in shorter-term clinical trials with solriamfetol, with headache, nausea, insomnia, nasopharyngitis, dry mouth, anxiety, decreased appetite, and upper respiratory tract infection as the most common TEAEs. Serious TEAEs were more frequent among participants with OSA (*n* = 21; 5.0%) than narcolepsy (*n* = 6; 2.7%); however, the OSA population comprised the larger portion of the safety database. Furthermore, the lack of a pattern in the occurrence, types, or timing of serious TEAEs, which ranged across system organ classes, suggests no unifying pathophysiology of a specific risk. Cardiovascular serious TEAEs were primarily reported in participants with OSA, which is consistent with the established high prevalence of cardiovascular comorbidities in that population [47]. Of note, the use of solriamfetol was not associated with a decrease in primary OSA therapy use over the course of the 12-week OSA study [37] or the current 1-year long-term extension study.

A major strength of this study was the large and somewhat heterogeneous sample (narcolepsy with and without cataplexy, OSA with varying levels of adherence to different primary OSA therapies). For the OSA population, this heterogeneity was reflective of patients seen in real-world clinical settings in that current or continuous use of a primary OSA therapy was not a requirement for participation. An additional strength was the inclusion of a double-blind, placebo-controlled RW period to demonstrate long-term maintenance of effect relative to placebo. However, there are several limitations, including that solriamfetol was not compared with other wake-promoting agents. Further research is needed to determine comparative effectiveness. Similarly, because the data were collected in the context of rigorously performed clinical trials, it is possible that the results reported here may differ from those observed in clinical practice. However, it should be noted that the OSA study population consisted of participants who were adherent and nonadherent to a primary OSA therapy, such as CPAP, providing a representation of the real-world population where adherence with CPAP therapy is variable [7]. It is important to evaluate the safety and efficacy of medications such as solriamfetol in patients who are nonadherent with primary OSA therapy given that these individuals are likely to be exposed to these treatments in practice. It is important to note that medications such as solriamfetol are not intended to replace primary OSA therapy; even for individuals treated with such medications, hypoxemia must be treated, and primary OSA therapy use should be strongly encouraged and supported with monitoring of such use. Another limitation is that this study focused on patient-reported outcomes and was not designed to assess objective outcomes such as the maintenance of wakefulness test (MWT). The MWT has been characterized in 12-week randomized controlled studies in both populations and has shown sustained efficacy [36, 37]. Moreover, neither neurocognitive performance nor motor vehicle accident risk resulting from EDS was assessed. However, it should be noted that motor vehicle accidents were captured as part of TEAEs, and that the six reported accidents that occurred were neither assessed as related to treatment nor appeared to be associated with sleepiness. Despite these limitations, the findings from this long-term study show that the sustained efficacy of solriamfetol is consistent with the efficacy observed in multiple randomized controlled clinical studies of shorter duration, and are likely to guide clinical practice in the future.

In conclusion, the long-term maintenance of efficacy with solriamfetol was demonstrated for the treatment of EDS in participants with narcolepsy or OSA. During the maintenance phase, improvements with solriamfetol were maintained for up to 1 year. Patients who completed approximately 6 months of treatment and remained on solriamfetol did not show loss of efficacy relative to those who were randomized to placebo. No rebound sleepiness, or withdrawal-related TEAEs were observed after abrupt discontinuation of solriamfetol during the RW phase. The safety profile was consistent with prior placebo-controlled studies of solriamfetol, and there were no safety concerns that emerged with chronic administration of up to 1 year.

## Supplementary Material

zsz220_suppl_Supplemental_Table_S1

zsz220_suppl_Supplemental_Figure
