# Supplementary material for: Long-term study of the safety and maintenance of efficacy of solriamfetol (JZP-110) in the treatment of excessive sleepiness in participants with narcolepsy or obstructive sleep apnea
Source: Sleep. 2019 Nov 6;43(2):zsz220. doi: 10.1093/sleep/zsz220 (PMC7315408; doi:10.1093/sleep/zsz220)
Supplement: zsz220_suppl_Supplemental_Table_S1 [file zsz220_suppl_supplemental_table_s1.docx]

**Supplemental Table S1. Serious TEAEs Across the Study (Safety Population, Groups A and B Combined)**

| System Organ Class Preferred Term | Number (%) of participants in combined solriamfetol groups | | |
| --- | --- | --- | --- |
|  | Overall (*N* = 643) | OSA (*n* = 417) | Narcolepsy (*n* = 226) |
| Participant with at least 1 serious event | 27 (4.2) | 21 (5.0) | 6 (2.7) |
| Cardiac Disorders | 4 (0.6) | 4 (1.0) | 0 |
| Acute myocardial infarction | 1 (0.2) | 1 (0.2) | 0 |
| Angina pectoris | 1 (0.2) | 1 (0.2) | 0 |
| Atrial fibrillation | 2 (0.3) | 2 (0.5) | 0 |
| Ear and Labyrinth Disorders | 1 (0.2) | 1 (0.2) | 0 |
| Vertigo | 1 (0.2) | 1 (0.2) | 0 |
| Eye Disorders | 1 (0.2) | 1 (0.2) | 0 |
| Retinal vein occlusion | 1 (0.2) | 1 (0.2) | 0 |
| Gastrointestinal Disorders | 3 (0.5) | 3 (0.7) | 0 |
| Abdominal pain | 1 (0.2) | 1 (0.2) | 0 |
| Duodenal ulcer hemorrhage | 1 (0.2) | 1 (0.2) | 0 |
| Gastrointestinal inflammation | 1 (0.2) | 1 (0.2) | 0 |
| Nausea | 1 (0.2) | 1 (0.2) | 0 |
| Vomiting | 1 (0.2) | 1 (0.2) | 0 |
| General Disorders and Administration Site Conditions | 3 (0.5) | 3 (0.7) | 0 |
| Chest discomfort | 1 (0.2) | 1 (0.2) | 0 |
| Chest pain | 1 (0.2) | 1 (0.2) | 0 |
| Noncardiac chest pain | 1 (0.2) | 1 (0.2) | 0 |
| Hepatobiliary Disorders | 1 (0.2) | 1 (0.2) | 0 |
| Cholecystitis acute | 1 (0.2) | 1 (0.2) | 0 |
| Immune System Disorders | 1 (0.2) | 0 | 1 (0.4) |
| Anaphylactic reaction | 1 (0.2) | 0 | 1 (0.4) |
| Infections and Infestations | 3 (0.5) | 3 (0.7) | 0 |
| Bronchitis | 1 (0.2) | 1 (0.2) | 0 |
| Cellulitis | 1 (0.2) | 1 (0.2) | 0 |
| Clostridium difficile infection | 1 (0.2) | 1 (0.2) | 0 |
| Sepsis | 1 (0.2) | 1 (0.2) | 0 |
| Staphylococcal infection | 1 (0.2) | 1 (0.2) | 0 |
| Injury, Poisoning, and Procedural Complications | 4 (0.6) | 3 (0.7) | 1 (0.4) |
| Alcohol poisoning | 2 (0.3) | 1 (0.2) | 1 (0.4) |
| Anemia postoperative | 1 (0.2) | 1 (0.2) | 0 |
| Ear canal injury | 1 (0.2) | 1 (0.2) | 0 |
| Fall | 1 (0.2) | 1 (0.2) | 0 |
| Head injury | 1 (0.2) | 1 (0.2) | 0 |
| Intentional overdose | 1 (0.2) | 0 | 1 (0.4) |
| Procedural hypotension | 1 (0.2) | 1 (0.2) | 0 |
| Skull fracture | 1 (0.2) | 1 (0.2) | 0 |
| Skull fractured base | 1 (0.2) | 1 (0.2) | 0 |
| Metabolism and Nutrition Disorders | 1 (0.2) | 1 (0.2) | 0 |
| Dehydration | 1 (0.2) | 1 (0.2) | 0 |
| Neoplasms Benign, Malignant, and Unspecified (Incl. Cysts And Polyps) | 2 (0.3) | 2 (0.5) | 0 |
| Malignant melanoma | 1 (0.2) | 1 (0.2) | 0 |
| Prostate cancer stage I | 1 (0.2) | 1 (0.2) | 0 |
| Nervous System Disorders | 5 (0.8) | 3 (0.7) | 2 (0.9) |
| Cerebrovascular accident | 1 (0.2) | 1 (0.2) | 0 |
| Cluster headache | 1 (0.2) | 0 | 1 (0.4) |
| Dizziness | 2 (0.3) | 2 (0.5) | 0 |
| Migraine | 1 (0.2) | 0 | 1 (0.4) |
| Pregnancy, Puerperium, and Perinatal Conditions | 2 (0.3) | 1 (0.2) | 1 (0.4) |
| Abortion spontaneous | 1 (0.2) | 0 | 1 (0.4) |
| Stillbirth | 1 (0.2) | 1 (0.2) | 0 |
| Psychiatric Disorders | 3 (0.5) | 1 (0.2) | 2 (0.9) |
| Agitation | 1 (0.2) | 0 | 1 (0.4) |
| Bipolar I disorder | 1 (0.2) | 1 (0.2) | 0 |
| Depression | 1 (0.2) | 0 | 1 (0.4) |
| Hallucination, auditory | 1 (0.2) | 0 | 1 (0.4) |
| Suicide attempt | 1 (0.2) | 0 | 1 (0.4) |
| Renal and Urinary Disorders | 1 (0.2) | 1 (0.2) | 0 |
| Hematuria | 1 (0.2) | 1 (0.2) | 0 |
| Reproductive System And Breast Disorders | 1 (0.2) | 1 (0.2) | 0 |
| Prostatomegaly | 1 (0.2) | 1 (0.2) | 0 |
| Respiratory, Thoracic, and Mediastinal Disorders | 1 (0.2) | 1 (0.2) | 0 |
| Pulmonary embolism | 1 (0.2) | 1 (0.2) | 0 |
| Vascular Disorders | 1 (0.2) | 1 (0.2) | 0 |
| Deep vein thrombosis | 1 (0.2) | 1 (0.2) | 0 |

OSA, obstructive sleep apnea; TEAEs, treatment-emergent adverse events.
