# Supplementary figures and images for: Long-term study of the safety and maintenance of efficacy of solriamfetol (JZP-110) in the treatment of excessive sleepiness in participants with narcolepsy or obstructive sleep apnea
Source: Sleep. 2019 Nov 6;43(2):zsz220. doi: 10.1093/sleep/zsz220 (PMC7315408; doi:10.1093/sleep/zsz220)

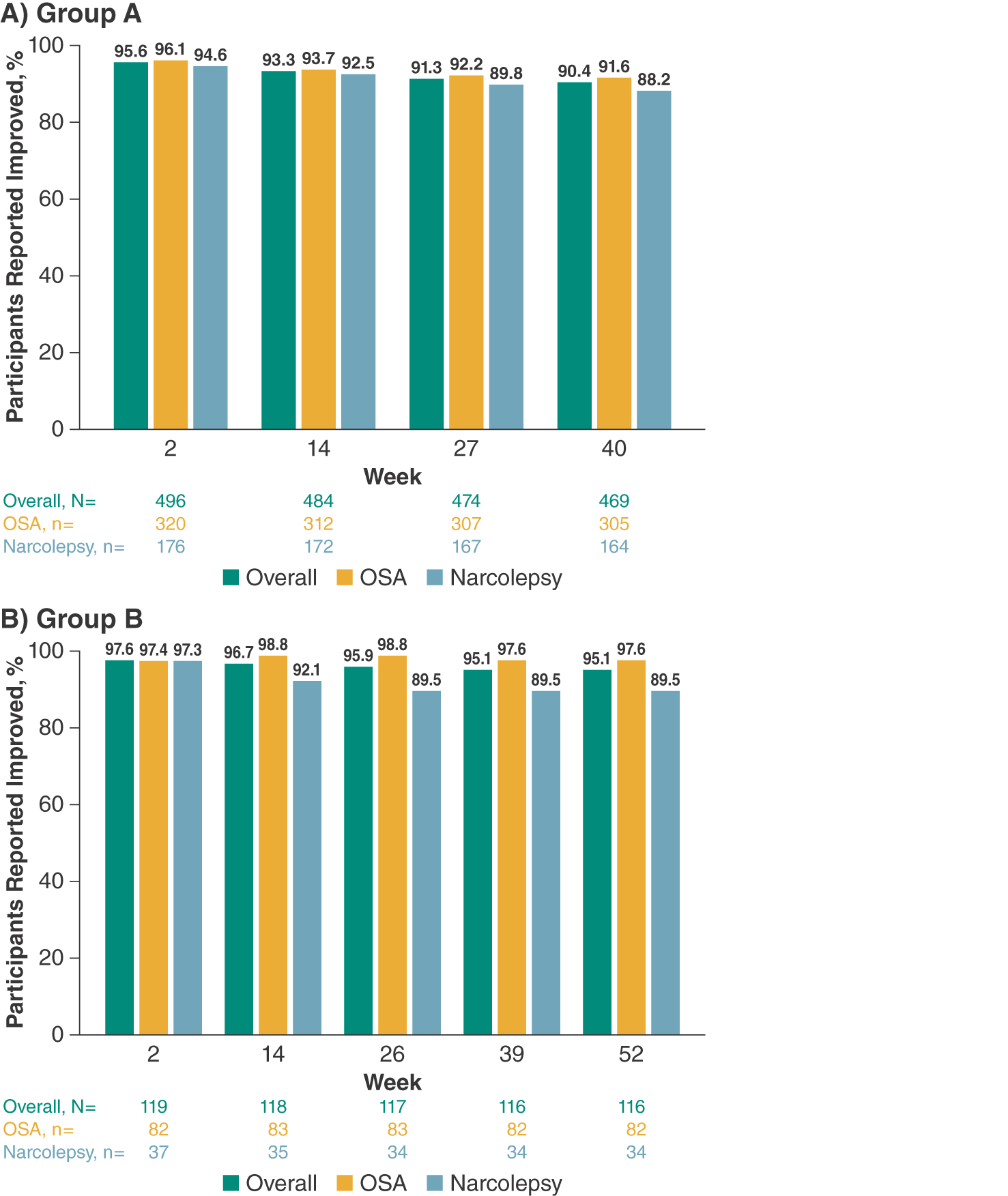

Supplement: zsz220_suppl_Supplemental_Figure [file zsz220_suppl_supplemental_figure.png]
